# Seroprevalence of brucellosis and associated factors among livestock slaughtered in Oko-Oba abattoir, Lagos State, southwestern Nigeria

**DOI:** 10.11604/pamj.2020.36.53.21094

**Published:** 2020-06-02

**Authors:** Kenneth Onyebuchi Ukwueze, Olayinka Olabisi Ishola, Magbagbeola David Dairo, Emmanuel Jolaoluwa Awosanya, Simeon Idowu Cadmus

**Affiliations:** 1 Nigeria Field Epidemiology and Laboratory Training Programme, Abudja, Nigeria; 2 Department of Veterinary Public Health and Preventive Medicine, Faculty of Veterinary Medicine, University of Ibadan, Ibadan, Nigeria; 3 Department of Medical Statistics and Epidemiology, Faculty of Public Health, College of Medicine, University of Ibadan, Ibadan, Nigeria

**Keywords:** Seroprevalence, *Brucella* infection, abattoir, Lagos State, livestock

## Abstract

**Introduction:**

*Brucella* infection, a neglected tropical zoonosis, poses public health threat to abattoir workers in developing countries including Nigeria. Oko-Oba abattoir is one of the largest abattoirs in the country that collects livestock from different parts of the country. This study determined the prevalence and factors associated with seropositivity of brucellosis among livestock slaughtered at Oko-Oba abattoir.

**Methods:**

A cross-sectional study was conducted from January to May 2018. A total of 473 serum samples were collected from livestock at the abattoir and tested for antibodies to *Brucella* species using the Rose Bengal Plate Test (RBPT) and indirect Enzyme Linked Immunosorbent Assay (iELISA). Data were analyzed using descriptive statistics and chi square test (p < 0.05).

**Results:**

Overall seroprevalence values were 15.3% (RBPT) and 16.3% (iELISA) among the livestock slaughtered at the Oko-Oba abattoir. Seroprevalence of 17.2% (RBPT) and 15.8% (iELISA) in cattle; 15.1% (RBPT) and 14.5% (iELISA) in goat; and 8.3% (RBPT) and 23.3% (iELISA) in sheep were obtained. Higher seroprevalence were recorded among females in cattle (18.8% iELISA) and sheep (23.1% iELISA) while male goats had average value higher (14.7% iELISA) than the female (p > 0.05).

**Conclusion:**

Presence of *Brucella* infection among slaughtered livestock was confirmed at Oko-Oba abattoir, Lagos State, Nigeria and poses a threat to abattoir workers and public health. Control of the disease in livestock and use of personal protective gear is recommended.

## Introduction

Brucellosis occurs worldwide but is better controlled in developed countries by routine screening of domestic animals and vaccination. The disease is a major neglected zoonosis of developing countries including Nigeria [1]. However, the prevalence varies in time and space among different livestock [2]. Brucellosis is endemic in subSaharan Africa, with significant effects on economic and social conditions of people in this region [3,4]. The incidence is influenced by management factors, herd size, population density, type of animal breed and biological features such as herd immunity [5, 6]. McDermott and Arimi [7] made a summary of data in cattle prior to 2001 which varied from 7.5% to 40% for pastoralists in arid and semiarid areas, 0.3-25.4% for cash/subsistence crops with livestock in sub-humid areas, 1.5-16.2% for crop-livestock in tropical highlands and 2.4-45.0% for crop with small-scale livestock production in humid areas. Studies reported seroprevalence of 2.9% with iELISA in argentine Creole sheep, 12.0% in Nepal and 4.1% to 6% in Costa Rica among cattle, respectively [8-10]. However in Ethiopia, 1.6% was reported in pastoral goats [11]. It is evidenced that the distribution and seroprevalence of brucellosis differs with species and location.

In Nigeria, several serological investigations have shown that *Brucella* infection is endemic in livestock population. In cattle, prevalence of 8.6% in Lagos State [12]; and 37.0% in three northern states (Kaduna, Kano and Adamawa) [13] have been reported. Also, another study in north-central Nigeria reported prevalence of 16.1% in cattle and most recent abattoir study in Ibadan, south-west Nigeria recorded a prevalence of 7.8% among cattle slaughtered at the abattoir [14]. Most of the investigation were done in cattle, although few documented evidence shows that the disease also exist in small ruminants, with prevalence of 0.86%, 14.0% and 25.80% reported in south-west, north-east and north-central Nigeria, respectively [15, 16]. Ogugua *et al*.[17] reported a prevalence of 2.83% among goats across four states in Nigeria (Borno, Oyo, Benue and Sokoto). However, the rates of *Brucella* infections recorded among cattle in these states is useful because it is a common practice for cattle and small ruminants to share common grazing and watering points in the north. This variation in prevalence may be due to geography, animal source, sample size, sampling technique and interpretation of tests [17]. Most importantly, a large number of animals in slaughter houses are culled by farmers for unproductiveness which could be indicative of diseases. The source and location of animals serve as potential risk factors in the epidemiology of brucellosis in animals. The widespread incidence of brucellosis could transmit to increase in human cases [4], especially among individuals at risk. There was therefore, need to undertake this evidence-based and multidisciplinary study of *Brucella* infection in different livestock slaughtered at an abattoir to evaluate the potential impact of *Brucella* infection and to inform effective control strategies. Hence, this study aimed at determining the seroprevalence of *Brucella* infection in livestock slaughtered at Oko-Oba abattoir and identifying possible risk factors.

## Methods

**Study design:** we conducted a cross sectional study to determine the prevalence and factors responsible for seropositivity of *Brucella* infection in sera of animals slaughtered at Oko-Oba abattoir, Lagos between January and May 2018.

**Study setting:** the study was at an abattoir facility in Oko-oba, Agege, Lagos State, Nigeria. Lagos State is bounded in the north and east by Ogun State, in the south by Atlantic Ocean and in the west by the Republic of Benin. The international boundary with the Republic of Benin is a unique opportunity for cross-border trading. It occupies a land area of 3,577 square kilometers [18]. The projected population of Lagos State from Nigeria´s 2006 National census is estimated to be 15 million [18]. About 75% of the population lives in urban areas. Oko-Oba abattoir ranked the largest abattoir in Nigeria and biggest in West Africa [19]. About 3000 livestock are slaughtered daily. It has an automated slaughtering line, rendering units and waste treatment plant [20]. The abattoir accounts for 50% of meat consumed in Lagos State.

**Study subjects:** the study population was made up of all livestock that were slaughtered at the Oko-Oba abattoir. Cattle, goats and sheep at the abattoir at the time of sample collection were included in the study while all cattle, goats and sheep at the abattoir whose owner did not consent at the time of sampling were excluded.

**Sample size consideration:** the sample size was determined based on prevalence of 8.6% [12], 2.83% [17] and 3.6% [21] *Brucella* infection reported for cattle, goats and sheep, respectively. The formula:

$$n=\frac{Z^{2}*pq}{d^{2}}$$

for sample size calculation in a cross sectional study was used. The reliability coefficient (Z) is 1.96 at 95% confidence level. The margin of error (d) of 4% was used for both cattle and sheep; while 2.5% was used for goats for good precision. All sample size calculations were adjusted for 10% non-response rate; this gave a sample size (n) of 215 cattle, 178 goats and 91 sheep. However, 221 cattle, 192 goats and 60 sheep (due to low number of sheep slaughtered throughout the study period) were used for the study.

**Data collection and collection of serum samples from the livestock:** three milliliters (ml) of venous blood was collected under strict hygienic conditions using sterile syringes and needles from cattle, goats and sheep just before slaughter into labeled sterile 5ml plain serum tubes and kept in slanted position on ice for serum formation. Serum samples were transported to the laboratory on ice for further processing. Each serum sample was labeled with a code that corresponds to the study site and subject identification. Intrinsic factors such as sex, age, body score and breed were recorded for each livestock species accordingly.

**Serological testing:** the Rose Bengal plate test (RBPT) consisting of standardized *Brucella abortus* and *Brucella melitensis* antigens sourced from the Animal Health and Veterinary Laboratories Agency, Weybridge U.K. was used for the *Brucella* test. Accurately, equal volumes (30μl) of antigen and test serum were mixed thoroughly on a plate using a spatula and the plate was rocked for 4 minutes. The appearance of agglutination within 2 minutes was scored 2+ (++), while the agglutination after 2 minutes was scored 1+ (+). The absence of agglutination after 4 minutes was scored negative (-). However, all cattle and sheep, and 179 of goat samples were further subjected to indirect enzyme linked immuno-sorbent assay (iELISA), using kit which was sourced from IDvet Innovative Diagnostics, Grabels, France; a multi-specie indirect ELISA diagnostic kit designed for detection of antibodies directed against *Brucella abortus* (bovine), *B. melitensis* (ovine and caprine) and *B. suis* (swine) in serum. The test was carried out according to the manufacturer´s procedure. The outcome was measured by ELISA reader and read as Optical Density (O.D.) values at 450nm. We defined a seropositive animal as any serum on screening for the presence of antibodies which tested positive to either RBPT or has sample / positive O.D. percentage of greater than or equal to 110% result by the iELISA. Seronegative animal is any animal whose serum was collected at the same time with the seropositive animal and on screening tested negative to RBPT or has a sample / positive O.D. of less than 110% result by the iELISA.

**Data analysis:** the age of the animal was determined using estimation of age by teeth appearance [22]. The body conditions were assessed by clinicians through examination and grading based on automatic grading of livestock [23]. Data were analyzed using Epi-Info version 7.2 software. Demographic characteristics were measured as the independent variables while the outcome of the serological test was measured as the dependent variables. We determined frequencies, proportions, and odds ratios. Chi square test was used to test level of significance in the values of the odds ratios at p < 0.05.

**Ethical considerations:** ethical approval for this study was obtained from the Lagos State Ministry of Agriculture. Permission was also obtained from the management of the abattoir where the study was conducted. Voluntary informed consent was explained in writing and obtained from the owners of each eligible animal before sample collection.

## Results

Of the 221 cattle sampled; most 154 (69.7%) were cows; and 81 (36.7%) were Rahaji breed, followed by Adamawa Gudali 37 (16.7%) and Ambala having the least 13 (5.9%). Most 218 (98.6%) of the cattle were adult, while 139 (62.9%) had good body condition. Of the 192 goats, the most breed 126 (65.6%) was Red Sokoto and 190 (98.9%) had good body condition. Of the 60 sheep, the most common breed 45 (75´0%) was Uda, and 51 (98.3%) were adults (Table 1). Overall seroprevalence of *Brucella* infection at the abattoir by Rose Bengal Plate Test (RBPT) was 15.3% (72 of 473); while by indirect Enzyme Linked Immuno-sorbent Assay (iELISA) was 16.3% (75 of 460). The seroprevalence of *Brucella* infection by RBPT in cattle, goats and sheep were 17.2%, 15.1% and 8.3%, respectively. By iELISA, *Brucella* infection seroprevalence of 15.8%, 14.5 % and 23.3% were obtained for cattle, goats and sheep, respectively (Table 2). There was no statistical difference (p > 0.05) in the *Brucella* infection seroprevalence among the species (Table 3). For breed-specific seroprevalence; the highest (33.3%) was among cross breed in cattle by RBPT; and 30.8% among Ambala breed by iELISA. In goats, the highest breed specific seroprevalence of 16.7% was among Red Sokoto by RBPT; and among Sahel breed (25.0%) by iELISA. In sheep, the Uda breed has the highest seroprevalence both by RBPT (8.8%) and iELISA (24.4%); the West African Dwarf (WAD) breed was negative for both tests (Table 2). None of the intrinsic factors such as sex, age, body score and breed in all the livestock species-cattle, goats and sheep was significantly associated with *Brucella* infection both by RBPT (Table 3) and iELISA (Table 4). However, in cattle, the Ambala breed was eight times more likely (Odds ratio: 7.7; 95% confidence interval: 1.2-49.2) to be *Brucella* positive by iELISA compared to the Adamawa Gudali (Table 4).

**Table 1:** Demographic characteristics of livestock slaughtered at Oko-Oba abattoir, Lagos, Nigeria

Variables	Cattle N =221(%)	Goat N = 192(%)	Sheep N = 60(%)
**Sex**			
Male	67 (30.3)	176 (91.7)	8 (13.3)
Female	154 (69.7)	16 (8.3)	52 (86.7)
**Age (Years)**			
Adult	218 (98.6)	190 (98.9)	59 (98.3)
Young	3 (1.4)	2 (1.1)	1 (3.3)
**Body Conditions**			
Good	139 (62.9)	190 (98.9)	42 (70.0)
Fair	69 (31.2)	2 (1.1)	13 (21.7)
Poor	13 (5.9)	*	5 (8.3)
**Breed**			
Adamawa Gudali	37 (16.7)	*	*
Bunaji	41 (18.5)	*	*
Ambala	13 (5.9)	*	*
Cross breed (Cattle)	24 (10.9)	*	*
Red Bororo (Rahaji)	81 (36.7)	*	*
Sokoto Gudali	25 (11.3)	*	*
Red Sokoto	*	126 (65.6)	*
Sahel	*	20 (10.4)	*
Cross breed (Goat)	*	46 (24.0)	*
Uda	*	*	45 (75.0)
Yankasa	*	*	13 (21.7)
West African Dwarf	*	*	2 (3.3)

NOTES: * = not applicable

**Table 2:** Seroprevalence of brucella infection in slaughtered livestock at Oko Oba Abattoir, Lagos, May 2018

RBPT (N =473)			iELISA (N = 460)	
Species	No of Samples	Seropositive (%)	No of Samples	Seropositive (%)
**Cattle: Breed**				
Adamawa Gudali	37	8 (21.6)	37	2 (5.4)
Bunaji	41	9 (22.0)	41	7(17.1)
Ambala	13	2 (15.4)	13	4 (30.8)
Cross breed (Cattle)	24	8 (33.3)	24	5 (20.8)
Red Bororo	81	9 (11.1)	81	15 (18.5)
Sokoto Gudali	25	2 (8.0)	25	2 (8.0)
Total	221	38 (17.2)	221	35 (15.8)
**Goat: Breed**				
Red Sokoto	126	21 (16.7)	117	17 (14.5)
Sahel	20	2 (10 .0)	20	5 (25.0)
Cross breed (Goat)	46	6 (13.0)	42	4 (9.5)
Total	192	29 (15.1)	179	26 (14.5)
**Sheep : Breed**				
Uda	45	4 (8.9)	45	11 (24.4)
Yankasa	13	1 (7.7)	13	3 (23.1)
West African dwarf	2	0 (0.0)	2	0 (0)
Total	60	5 (8.3)	60	14 (23.3)

**Table 3:** Intrinsic factors associated with seroprevalence of Brucella infection using rose bengal plate test among livestock slaughtered at Oko oba abattoir, Lagos

Variables	No of samples	+ve (%)	OR (CI)	P-value
**Species (n = 473)**				
Cattle	221	38 (17.2)	1	
Goat	192	29 (15.1)	0.9 (0.5-1.5)	0.57
Sheep	60	5 (8.3)	0.4 (0.2-1.2)	0.09
**Species: Cattle (n= 221)**				
**Sex**				
Male	67	7(10.5)	2.2 (0.8-5.2)	0.08
Female	154	31(20.1)		
**Age**				
Adult	218	37(17.0)	0.4 (0.0-4.6)	0.46
Young	3	1 (33.3)		
**Body**				
Good	139	26 (18.7)	1	
Fair	69	8 (11.6)	1.8( 0.7-4.1)	0.19
Poor	13	4 (30.8)	0.5 (0.1-1.8)	0.30
**Breed**				
Adamawa Gudali	37	8 (21.6)	1	
Ambala	13	2 (15.4)	0.7 (0.1-3.6)	0.63
Bunaji	41	9 (22.0)	1.0 (0.3-2.9)	0.97
Cross breed (cattle)	24	8 (33.3)	1.8 (0.6-5.8)	0.31
Red Bororo	81	9 (11.1)	0.5 (0.2-1.3)	0.13
Sokoto Gudali	25	2 (8.0)	0.3 (0.1-1.6)	0.16
**Species: Goat (n = 192)**				
**Sex**				
Male	176	26(14.8)	1.3 (0.4-5.0)	0.67
Female	16	3 (18.7)		
**Age**				
Adult	190	28(14.7)	0.2 (0.0-2.8)	0.17
Young	2	1 (50.0)		
**Body**				
Good	190	29 (15.3)	1	
Fair	2	0 (0.0)	na	0.55
Poor	0	0	-	-
**Breed**				
Cross breed (Goat)	46	6 (13.0)	1	
Red Sokoto	126	21 (16.7)	1.3 (0.5-3.5)	0.56
Sahel	20	2 (10.0)	0.7 (0.1-4.0)	0.73
**Species: Sheep (n = 60)**				
**Sex**				
Male	8	0 (0.0)	na	0.36
Female	52	5(9.6)		
**Age**				
Adult	59	5 (8.5)	na	0.76
Young	1	0 (0.0)		
**Body**				
Poor	5	0 (0.0)	1	
Fair	13	2 (15.4)	na	0.37
Good	42	3 (7.1)	na	0.54
**Breed**				
Uda	45	4 (8.9)	1	
Yankasa	13	1 (7.7)	0.9 (0.1-8.4)	0.89
West African dwarf	2	0 (0.0)	0.0 (0.0-1.0)	0.97

NOTES: na = undefined value, +ve = Positive, OR = Odd ratio, CI = Confidence interval

**Table 4:** Intrinsic factors associated with seroprevalence of Brucella infection using iELISA among livestock slaughtered at Oko Oba abattoir, Lagos

Variables	No of samples	+ve (%)	OR (CI)	P-value
**Species (n = 460)**				
Cattle	221	35 (15.8)	1	
Goat	179	16 (14.5)	0.9 (0.5-1.6)	0.71
Sheep	60	14 (23.3)	1.6 (0.8-3.3)	0.17
**Species: Cattle (n = 221)**				
Sex				
Male	67	6 (9.0)	2.4 (0.9-6.0)	0.07
Female	154	29 (18.8)		
**Age**				
Adult	218	35(16.1)	Na	0.45
Young	3	0 (0.0)		
**Body**				
Good	154	19 (13.7)	1	
Fair	69	15 (21.7)	0.6 (0.3-1.2)	0.14
Poor	13	1 (7.7)	1.9 (0.2-10. 5)	0.54
**Breed**				
Adamawa Gudali	37	2 (5.4)	1	
Ambala	13	4 (30.8)	7.7 (1.2-49.2)	0.03**
Bunaji	41	7 (17.1)	3.6 (0.7-18.5)	0.12
Cross breed (cattle)	24	5 (20.8)	4.6 (0.8-25.9)	0.08
Red Bororo	81	15 (18.5)	3.9 (0.9-18.3)	0.77
Sokoto Gudali	25	2 (8.0)	1.5 (0.2-11.5)	0.69
**Species: Goat (n = 179)**				
**Sex**				
Male	163	24 (14.7)	0.8 (0.2-3.9)	0.81
Female	16	2 (12.5)		
**Age**				
Adult	177	26 (14.7)	Na	0.56
Young	2	0 (0.0)		
**Body**				
Good	177	26 (14.7)		
Fair	2	0 (0.0)	Na	0.56
Poor	0	0 (0.0)	-	-
**Breed**				
Cross breed (Goat)	42	4 (9.5)	1	
Red Sokoto	117	17 (14.5)	1.6 (0.5-5.1)	0.41
Sahel	20	5 (25.0)	3.2 (0.7-13.4)	0.11
**Specie: Sheep (n = 60)**				
**Sex**				
Male	8	2 (25.0)	1.1 (0.2-6.2)	0.90
Female	52	12 (23.1)		
**Age**				
Adult	59	14 (23.7)	Na	0.58
Young	1	0 (0.0)		
**Body**				
Poor	5	1 (20.0)	1	
Fair	13	2 (15.4)	0.7(0.5-10.4)	0.82
Good	42	11 (26.2)	1.4 (0.1-14.1)	0.76
**Breed**				
Uda	45	11 (24.4)	1	
Yankasa	13	3 (23.1)	0.9 (0.2-3.9)	0.98
West African dwarf	2	0 (0.0)	0.0 (0.0-1.0)	0.92

NOTES: na = undefined value, +ve = Positive, OR = Odd ratio, CI = Confidence interval,

** Significant at p < 0.05

## Discussion

The study confirmed that Brucella infection is present in cattle, goats and sheep slaughtered for consumption at Oko-Oba abattoir, Lagos and that seroprevalence varied across species, breed, sex and age of animals. In this study, two serological tests were adopted to confirm the seroprevalence of the livestock using serum samples. This method was also adopted by Kashiwazaki *et al.* [24] who demonstrated that combination of RBPT for screening of infected herds and the iELISA for identifying infected individuals was considered to be a quite appropriate and effective diagnostic tool for large scale serological survey of brucellosis. The RBPT is susceptible to cross reaction with other gram negative bacteria such as Yersinia enterocolitica O:9, Eschrichia coli O:157; and some Salmonella species, which could lead to false positive results [25]. Nonetheless, these two methods are suitable for the detection of Brucella antibodies in cattle and small ruminants. The study revealed an overall prevalence of 15.2% and 16.3% with the RBPT and iELISA, respectively among the livestock slaughtered at the abattoir. This was much higher than 2.6% reported among livestock in Niger [21] and 5.8% recorded by Assenga *et al.* in Tanzania [26]. A seroprevalence of 17.2% and 15.8% was observed among slaughtered cattle based on RBPT and iELISA, respectively. This is higher than the prevalence of 8.6% earlier reported among cattle in Lagos by Cadmus [12] and much higher than 4.9% reported by Ogugua *et al.* [27] in South Western Nigeria and 5.45% recorded by Bwala *et al.* [19] in Ibadan municipal abattoir Nigeria. Although, it was lower than 36.6% recorded among cattle in three northern states [13]. However the seroprevalence obtained in this study is much higher compared to other reports from developing countries which reported 2.21% in South Africa [28], and 2.90% in Ethiopia [29]. The difference in prevalence between our study and other previous studies could be partly due to the methodology used, in other previous studies only samples positive based on RBPT were tested with ELISA while our study tested all the samples with iELISA, the sampling frame, this frame was based on the number of livestock seen at time of sampling and may not depict the actual sampling frame of livestock slaughtered at the abattoir and also the study location the abattoir is the largest in the south west Nigeria and collects livestock from various locations across the country. Another important issue is the difference in sensitivity and specificity of serological tests used for screening. This factor contributes to the variations in results among researchers [30]. In small ruminants, seroprevalence of 15.1% and 14.5% was recorded in goat using RBPT and iELISA, respectively, this is quite high compared to previous reports which recorded 2.8% across four States in Nigeria [17]. It is also much higher than prevalence of 2.8% and 1.6% recorded in Somalia and Ethiopia respectively [11]. The likely reasons for the differences could also be due to the fact that the study was conducted in an abattoir which collects livestock from various locations across the country. Seroprevalence of 8.3% and 16.3% was observed among sheep using RBPT and iELISA, respectively. This is higher compared to prevalence of 3.6% [21] and 0.0% [15] reported by these authors. Most of these studies were conducted among herd in the farm, while this study was conducted in an abattoir. The remarkable difference in the prevalence of livestock surveyed is a reflection of the fact that quite a large number of animals in slaughter houses are culled by farmers due to poor performance. The similarity in the rate of infection recorded among the slaughtered livestock shows that trade animal record higher seropositivity compared to those kept in the farms [17].

The explainable reason for higher seroprevalence recorded in the slaughtered livestock could likely be due to the fact that most of the animals slaughtered in the abattoir are gotten from the livestock market and farmers are known to sell animals that are sick or unproductive [31]. More so, there is no prevention and control strategy adopted in Nigeria, contributing to the higher seroprevalence when compared to other studies in other climes where control strategy exists. Higher prevalence of the disease was demonstrated in cattle than in the small ruminants, though the variation is not statistically significant. Similar results were found in Nigeria in which the seroprevalence of the disease was higher in cattle than in goats [15]. The results are also similar to a study carried out in Tanzania which demonstrated higher prevalence in cattle than goats [26]. Although bacteriological analysis was not conducted to determine the strain of Brucella species that was detected in these livestock, the serological test with RBPT was B. abortus and B. melitensis antigen for cattle and small ruminant respectively, B. melitensis is the most pathogenic [32]. However, iELISA test is regarded as the confirmatory serological test as documented by Lopez *et al.* [33]. The seroprevalence recorded in this study suggests that the risk of transmission of brucellosis to abattoir workers is very high. The seroprevalence was more in female cattle than male; however there was no association with Brucella infection. Although most goat sampled were male, the study found high seropositive cases among female goats than male by RBPT; however, there was no association. By iELISA, male goats had higher seroprevalence than the female. In sheep higher prevalence was recorded in female than male by RBPT, but by iELISA, male sheep had higher seroprevalence than female, however this association was not statistically significant. This can be partly explained in terms of sample distribution, most of the sheep sampled were female. Males are kept separately where they are fed well for market value. Their market value is much higher than that of females and they are usually sold when there is a need for cash or are slaughtered during religious ceremonies [34]. Interestingly, it is of importance to note that earlier reports have shown that female animals are more susceptible to Brucella infection and also represents a greater risk of spreading infection [35,36] because they stay longer in the farm and are only culled when they become unproductive. Therefore, having more female animals in the abattoir predisposes the workers to Brucella infection as farmers are known to sell off their female animals which are not doing well, and the most important indicator of not doing well in female animals are reproductive failure and low milk production [37], which are clinical signs of brucellosis in animal.

In this study, the seroprevalence was higher in the adult than the young animals with both serological tests, although this was not statistically significant. This finding is similar to the report of Cadmus *et al.* [38], and Assenga *et al.* 26, but in contrast to Ogugua *et al.* [17] who reported high seroprevalence in young animals. This could be due to increase in concentration of sex hormone which stimulate multiplication of Brucella organism [36]. Again it could also be as a result of the younger animals being resistant to infection, and being able to clear this infection frequently, although re-infection could occur at older age similar to the findings of Ogugua *et al.* 17. Again, body condition could be a contributing factor to the differences in seroprevalence in livestock [31], on the contrary, this study found no statistical significant association in all the species surveyed. This study also highlights the variations observed in breed specific seroprevalence, in cattle, cross breed recorded the highest prevalence using RBPT, while Adamawa Gudali had the least. With iELISA, the highest breed specific was recorded in Ambala, this was in contrast to the findings of Ogugua *et al.* [38] who reported highest prevalence in Kuri and the reports of Cadmus *et al.* and Junaidu *et al.* [38,39] where the highest prevalence was reported in Bunaji and Sokoto Gudali, respectively. Although one of the factors implicated in conferment of resistance and tolerance is the genetic factor [40]. However, in cattle, the Ambala breed was eight times more likely to be Brucella positive by iELISA compared to the Adamawa Gudali, this shows that breed is associated with brucellosis among cattle in Nigeria [31]. Furthermore, the study found no significant association in both serological tests among goat and sheep breeds. Though, the highest seroprevalence was recorded among Red Sokoto and Uda using RBPT and iELISA, respectively; this is consistent with the reports of Ogugua *et al.* and Junaidu *et al.* [17,41] who reported highest prevalence in Red Sokoto with no significant association between seropositivity and breed of animal, This can equally be explained in terms of sample distribution, most of the goats sampled were Red Sokoto.

## Conclusion

Despite the varied seroprevalence of brucellosis in livestock found in the abattoir, these findings confirmed the endemicity of Brucella infection among livestock animals in Nigeria. There is need to establish control strategy for brucellosis among livestock animals by the Federal Ministry of Agriculture. More so, the potential threat the endemicity of brucellosis in animals poses to abattoir workers and other at risk persons calls for the heightened surveillance for brucellosis among febrile patients by the Federal Ministry of Health. A “One Health” collaborative approach in tackling the Brucella problem is hereby recommended.

### What is known about this topic

Brucellosis is endemic in sub-Saharan Africa, with major public health importance;

Brucellosis is a major neglected zoonosis affecting animals and humans causing abortion and other health conditions;

Serological methods including RBPT and indirect ELISA techniques have been used successfully in the epidemiological investigations of Brucella infections and brucellosis in animals.

### What this study adds

This study confirmed the presence of Brucella infection in livestock slaughtered in a major abattoir, Lagos State, Nigeria and that seroprevalence varied across species, breed, sex, and age of animals;

This study showed moderately high seroprevalence of Brucella infections of 17.2%, 15.1% and 8.3% by RBPT, and 15.8%, 14.5% and 23.3% by indirect ELISA among cattle, goats and sheep, respectively slaughtered in a major abattoir, Lagos State, Nigeria;

The Ambala breed of cattle was found to be more

susceptible to Brucella infection being eight times more likely to be Brucella positive compared to the Adamawa Gudali by iELISA.

## Competing interests

The authors declare no competing interests.
